# The Long-Lasting Story of One Sensor Development: From Novel Ionophore Design toward the Sensor Selectivity Modeling and Lifetime Improvement

**DOI:** 10.3390/s21041401

**Published:** 2021-02-17

**Authors:** Larisa Lvova, Donato Monti, Corrado Di Natale, Roberto Paolesse

**Affiliations:** 1 Department of Chemical Science and Technologies, University of Rome “Tor Vergata”, 00133 Rome, Italy; roberto.paolesse@uniroma2.it; 2 Department of Chemistry, La Sapienza University of Rome, 00185 Rome, Italy; donato.monti@uniroma1.it; 3 Department of Electronic Engineering, University of Rome “Tor Vergata”, 00133 Rome, Italy; dinatale@uniroma2.it

**Keywords:** co-porphyrin ligand, solvent polymeric membranes, J-type porphyrin aggregation, nitrite sensing

## Abstract

The metalloporphyrin ligand bearing incorporated anion-exchanger fragment, 5-[4-(3-trimethylammonium)propyloxyphenyl]-10,15,20-triphenylporphyrinate of Co(II) chloride, CoTPP-N, has been tested as anion-selective ionophore in PVC-based solvent polymeric membrane sensors. A plausible sensor working mechanism includes the axial coordination of the target anion on ionophore metal center followed by the formed complex aggregation with the second ionophore molecule through positively charged anion-exchanger fragment. The UV-visible spectroscopic studies in solution have revealed that the analyte concentration increase induces the J-type porphyrin aggregation. Polymeric membranes doped with CoTPP-N showed close to the theoretical Nernstian response toward nitrite ion, preferably coordinated by the ionophore, and were dependent on the presence of additional membrane-active components (lipophilic ionic sites and ionophore) in the membrane phase. The resulting selectivity was a subject of specific interaction and/or steric factors. Moreover, it was demonstrated theoretically and confirmed experimentally that the selection of a proper ratio of ionophore and anionic additive can optimize the sensor selectivity and lifetime.

## 1. Introduction

The success of new chemical sensors’ development in very high degree depends on the tailored choice of an appropriate ligand/ionophore responsible not only for selective analyte binding, but also determining the sensor stability, reproducibility, and lifetime. Among anion-selective ionophores metalloporphyrins are most widely used due to their rich electrochemical and optical properties and high structural versatility, which can be modulated both through the incorporation of various main-core substituents and/or through different central metal selections [1,2]. In fact, metalloporphyrin-based chemical sensors, and potentiometric ion selective electrodes (ISEs), in particular, are popular devices for specific detection of anions since they demonstrate the selectivity, different from the lyotropic Hofmeister series, determined by energies of hydration of target analyte [3]. The mechanism of metalloporphyrins’ anionic sensitivity is well established and occurs through selective axial coordination of a target anion on central metal ion [4,5]. However, metalloporphyrin-based ISEs present also several drawbacks, among them a hydroxide ion-bridged ionophore dimerization, which results in a non-Nernstian electrode behavior coupled to an unstable response, pH side effects, and short lifetime [6,7]. In the literature, several attempts to avoid the ionophore dimerization have been presented, among them an application of picket-fence porphyrins in which the steric hindrance prevents ionophores’ dimerization within the membrane phase, thus leading to theoretical response slopes [6,8] application of different types of polymeric matrices (other than PVC), such as silicone rubber [9], polyurethane [10,11], or direct porphyrins’ electropolymerization/copolymerization onto the working electrode surface [12,13,14,15,16,17,18]. The covalent attachment of the porphyrin ionophore to the various supports and further incorporation into polymeric matrices, for instance, polymethacrylate [19,20] or Single-Walled Carbon Nanotubes (SWCNTs) [21], the exploitation of highly lipophilic metalloporphyrin ionophores bearing bulky side moieties, as for instance, Ge(IV)-tert-butyl-tetraphenylporphyrin, Ge(IV)-tBTPP, reported in [22], or use of Pt-porphyrin ionophores having a coordinated metal with a reduced oxophylicity [23,24], were also studied and permitted to develop the anion-selective potentiometric membrane electrodes and optodes with improved characteristics. Although the abovementioned approaches provided the promising results, the problem of metalloporphyrin dimerization in the membrane phase resulting in the non-Nernstian and sluggish response toward analyte anion has not been completely solved. 

Previously, we decided to use in our investigations an opposite strategy based on ionophore and analyte co-aggregation promotion inside a membrane, with the final aim of the selectivity improvement toward a target analyte [25]. That time, the new porphyrinic complexes bearing lipophilic tris-methylammoniumpropyloxy fragment in para-position of 5,10,15,20-tetraphenylporphyrin complexes, MeTPP-N, of several metals were synthesized and tested in our laboratories [26]. These complexes underwent a spontaneous aggregation from aqueous solutions forming porphyrin films with an ordered morphology on the glass support. The aggregation mechanism was investigated in detail with spectroscopic techniques including combined resonance light scattering (RLS) studies and kinetic experiments, as well as with Atomic Force Microscopy (AFM) films’ morphology studies. The UV-vis spectral patterns of the films varied significantly in the presence of amines and olefins vapors, thus indicating their utility for chemical sensing. The UV-vis spectra variations were attributed to the aggregate’s macrostructure reorganization due to the inclusion of the analyte molecules within the layered porphyrin macrocycles through coordination on central metal and consecutive decrease of the π–π electronic interaction among the porphyrin chromophores. On the basis of these results, we decided to study the role of the analyte on MeTPP-N complexes’ aggregation inside a solvent polymeric membrane and to explore these molecules as membrane-active components with a challenge to develop novel sensing materials for liquid-phase analysis. 

In particular, we describe here the development steps of anion-selective potentiometric sensor, based on PVC solvent polymeric membranes doped with Co(II) tetraphenyl porphyrin derivative, CoTPP-N, through over 10 years’ period of investigations. The exploitation of MeTPP-N ligands in polymeric membrane sensors is of particular interest, since they possess two active centers inside the same molecule: a central metal as neutral or charged ligand site and the tris-methylammonium anion-exchanging site, that may additionally modulate the sensor selectivity. Moreover, being the big-size lipophilic molecules, MeTPP-N complexes have the sterically difficult leaching from the membrane phase, thus increasing the sensor lifetime. Finally, these compounds provide more possibilities of anionic selectivity tailoring, for instance, through the variation of central metal nature, by changing the length of aliphatic tether introducing the appended cationic functionality, and by incorporation of additional peculiar membrane-active components inside the MeTPP-N-based membrane (anion-exchanging sites, additional ionophore, specific plasticizer, etc.). Through our experiments, the membranes doped with new CoTPP-N ionophore demonstrated a selective Nernstian behavior toward nitrite ion in all the tested concentration ranges. A plausible working mechanism, including the axial coordination of the target anion on central metal of metalloporphyrin ligand, followed by subsequent coordination of the second ionophore molecule through an opposite molecular site bearing positively charged anion-exchanger fragment, was suggested. The UV-visible spectroscopic studies in several organic solvents and inside PVC membranes showed the formation of head-to-tail J-type porphyrin aggregates, thus supporting the suggested ionophore working mechanism. The only, but crucial, drawback of a developed sensor was a very short sensor lifetime: In practice the sensor was almost disposable, and the complete response deterioration was observed after its first utilization. The results of this study were reported on Matrafured-2008 International Conference on Electrochemical sensors [25]. After the very useful discussions with electrochemist colleagues, the further studies, consisting of numerous membrane composition optimizations, were conducted. In particular, the tests on incorporation of cationic or anionic lipophilic sites, as far as the membrane doping with additional 5,10,15,20-tetraphenylporphyrinate of Co(II), CoTPP, ionophore sites, were performed in order to keep high selectivity and achieve the long-lasting sensors’ stability. We reported the progress in our studies on ICPP-9 (International Conference on Porphyrins and Phthalocyanines) [27], describing the influence of ionophore aggregation on sensor response through the mathematical simulation of the sensor sensitivity. The obtained results were in accordance with the obtained experimental data. The performed modifications of membrane compositions permitted keeping high selectivity and achieving the long-lasting stability of the sensors containing CoTPP-N ligand. As a result of our research of more than a decade, the optimum composition of PVC-based solvent polymeric membrane nitrite-selective electrode, with a slope of −57.8 ± 3.2 mV/dec in the concentration range from 10^−6^ to 5 × 10^−2^ M, low detection limit (LDL) of 37 µM, and over one-month-long reproducible performance, was developed. Actually, the tests on MeTPP-N-based anion-selective potentiometric sensors and, in particular, on ZnTPP-N-doped sensors for selective detection of hydrophilic sulfate ions, are in progress in our laboratories, and so, it is not the end of the story: The research continues!

## 2. Experimental

### 2.1. Reagents

**Scheme 1:**
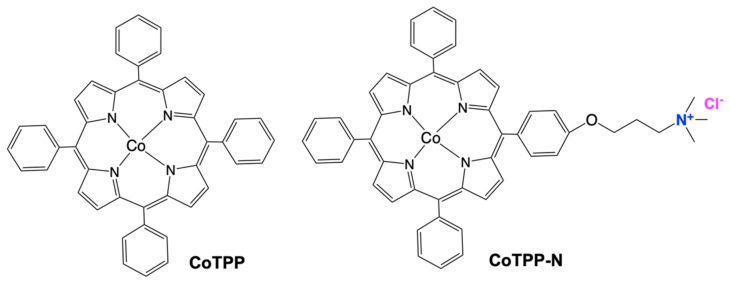
Chemical structure of the Co(II)-porphyrin ligands studied in the work.

Poly(vinyl chloride) (PVC) high-molecular-weight, o-nitrophenyl octyl ether (o-NPOE), potassium tetrakis-(4-chlorophenyl)borate (TpClPBK) and tridodecylmethyl-ammonium chloride (TDMACl) were purchased from Fluka. The ligands Co(II)-5-[4-(3-tris-methylammonium) propyloxyphenyl]-10,15,20-triphenyl-porphyrin chloride, CoTPP-N, and Co(II)- 5,10,15,20-tetraphenylporphyrin, CoTPP, were synthesized in our laboratories, according to the previously reported methods [26,28], and fully characterized. The chemical structures of tested ligands are shown in Scheme 1. Dichloromethane (CH_2_Cl_2_), acetonitrile (ACN), and tetrahydrofuran (THF) solvents, tetrabutylammonium nitrate (TBANO_2_) and 4-morpholinoethanesulfonic acid (MES), were purchased from Sigma-Aldrich. THF was freshly distilled prior to use. All the other chemicals were of analytic grade and used without further purification. The sample solutions for potentiometric measurements were prepared dissolving sodium salts of given anions in 0.01M MES pH 5.5 buffer background. 

### 2.2. Membranes’ Preparation and Evaluation

The membranes of 100-mg weight contained 1 wt.% of ionophore, PVC/oNPOE plasticizer (1:2) polymeric matrix, and varying amounts of lipophilic additives. The compositions of tested membranes are listed in Table 1. All components were dissolved in 1 ml of THF, stirred vigorously, and then about 10 μL of the membrane cocktails were cast on Glassy Carbon working electrode (GC WE) surface. Solvent was allowed to evaporate overnight. The GC WEs had a flat surface of 3 mm in diameter, 3 cm in height, and were incorporated in a Teflon body (AMEL, Italy). Before membrane casting, GC electrodes were polished with alumina slurries, cleaned for 15 min in ultrasonic bath, rinsed with ethanol, and dried on air. For spectroscopic studies, the 10 μL of membrane Mb1 cocktail in THF was deposited on the transparent glass slide (3 cm in length and 7 mm in width) by drop-casting method: The membrane spot of 5 mm diameter was well adhered to the glass surface after solvent evaporation. The freshly prepared electrodes were soaked in 0.01 M NaNO_2_ solution. The modified-with-membrane Mb1 glass slides for spectroscopic tests were conditioned also in 0.01M NaCl, when required (see sections below for details). Potentiometric properties of electrodes were evaluated one day after preparation. The measurements were performed in triplicate for every membrane formulation. Electrochemical potentials were measured against Saturated Calomel (SCE) reference electrode (AMEL, Italy) at ambient temperature (+22 °C) via a high-impedance, analog-to-digital potentiometer (LiquiLab, ECOSENS srl, Italy). Selectivity coefficients were calculated by the separate solution method (SSM) using membranes’ potential values measured at anion concentration of 0.01 M. The ions’ activities (log a) were calculated according to the second approximation of Debye–Hückel approach.

### 2.3. Kinetic Studies

UV-vis spectra were acquired on a Carry 50 spectrophotometer in a glass cell (1-cm path length). For membrane films’ testing, the glass slides with deposited polymeric membrane were preliminary soaked in 0.01 M solutions of NaNO_2_ or NaCl or, subsequently, in both of them for period of time from 2 h to 48 h. Kinetic experiments were performed at room temperature (25 °C) by measuring the UV-visible spectroscopic changes of CoTPP-N in the presence of different concentrations of TBANO_2_ with time. For this, CoTPP-N solutions of 5 × 10^−6^M concentration in ACN and CH_2_Cl_2_ containing TBANO_2_ were prepared as follows. The calculated aliquot of a 0.1 mM stock solution of CoTPP-N was added to 2.0 mL of corresponding solvent directly in the glass cuvette. To this solution the calculated amount of 1 mM TBANO_2_ stock solution (in ACN or CH_2_Cl_2,_ respectively) was then added, bringing the volume of a final solution to 3 mL. The relative UV-visible spectra were acquired in a time drive scan mode. The concentration of TBANO_2_ in tested solutions varied in the range from 1.0 × 10^−5^ to 5.0 × 10^−4^ M, spanning over 1.5 orders of magnitude. The kinetic parameters were obtained by nonlinear least-squares regression fitting by using a Kaleidagraph program (Synergy Software, 2007) over hundreds of experimental data points. Experiments were run in triplicate. 

## 3. Results and Discussion

### 3.1. Kinetic and Spectroscopic Studies on the Aggregation of Co(II)-5-[4-(3-Tris-Methylammonium) Propyloxyphenyl]-10,15,20-Triphenyl-Porphyrin Chloride in Solution

**Figure 1:**
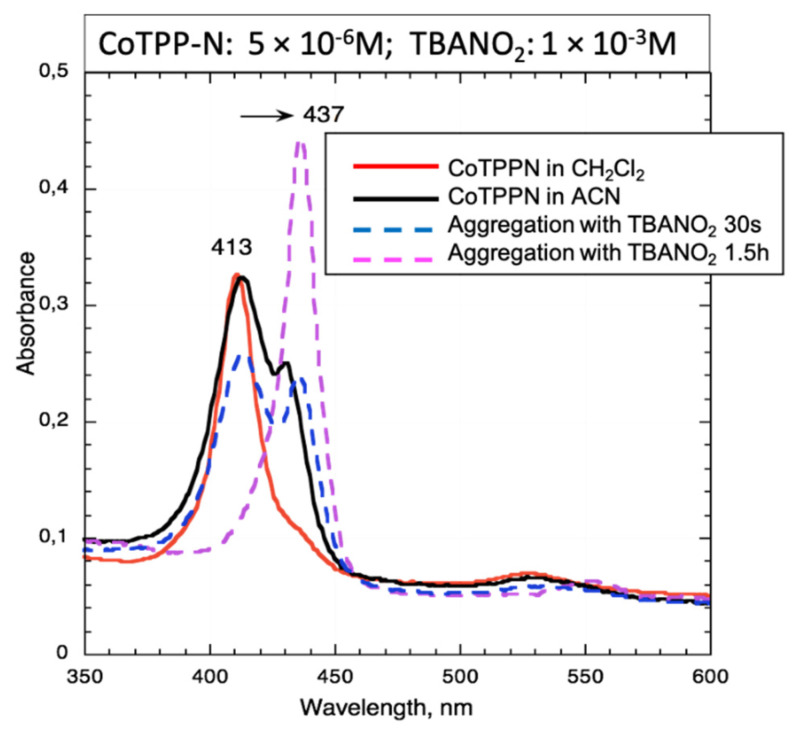
UV-vis spectra of CoTPP-N (5 × 10^−6^M) in CH_2_Cl_2_ (red line) and acetonitrile, ACN (black line). Dashed lines represent the evolution of CoTPP-N spectra in ACN in the presence of 1 mM TBANO_2_.

According to the literature data, the porphyrinic complexes of cobalt are known for the high affinity to nitrogen-bearing anions, and to nitrite ions, in particular [29]. Hence, we decided to study the interaction of Co(II)-5-[4-(3-trimethylammonium) propyloxyphenyl]-10,15,20-triphenyl-porphyrin and NO_2_^−^ ions, first in solution. The aggregation experiments were carried out in 5 × 10^−6^ M solutions of CoTPP-N in two different solvents, acetonitrile and dichloromethane at 25 °C. As shown in Figure 1, the sharp Soret band positioned at 413 nm on UV-visible spectra of CoTPP-N solution in CH_2_Cl_2_ evidences the presence of macrocycle in this solvent, mainly in monomeric form. A formation of a small shoulder at 430 nm in acetonitrile solvent could be attributed to the axial coordination of solvent on porphyrin co-center. Upon the addition of 1 mM TBANO_2_ to the CoTPP-N in ANC, a small bathochromic shift of 7 nm due to the axial coordination of NO_2_^−^ ion on CoTPP-N was initially observed. The further formation of CoTPP-N porphyrin aggregates was clearly evidenced by the growth of enlarged Soret band at 437 nm and decrease of monomer peak at 413 nm. Moreover, the presence of a well-defined isosbestic point indicated the occurrence of a controlled self-assembly process toward the formation of a narrow distribution of species. The formed porphyrin aggregates, probably with an initial head-to-tail morphology (i.e., of J-type structure), formed completely over the 1.5 h time to give assemblies of determined dimensions. 

**Figure 2:**
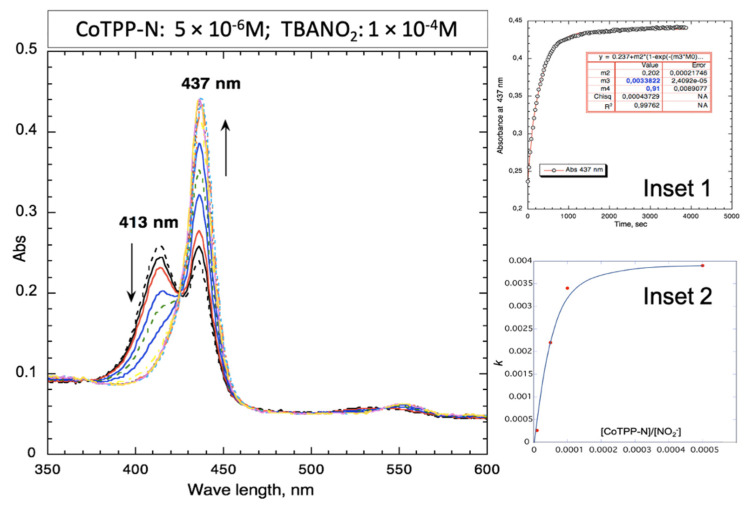
The evolution of UV-vis spectra of CoTPP-N (5 × 10^−6^M) in ACN, upon the aggregation in the presence of 1 × 10^−4^M TBANO_2_. Uppermost trace: t = 0, lowermost trace: t = 1.5 h. Inset 1: corresponding kinetic profile at wavelength of 437 nm. Inset 2: variation of the aggregation rate (*k_app)_* with [CoTPP-N]/[NO_2_^−^] ratio.

**Figure 3:**
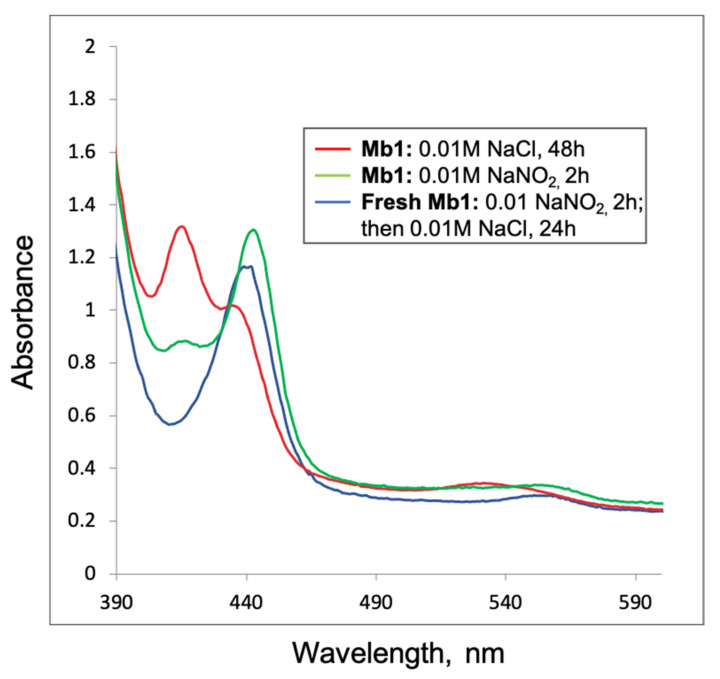
The UV-visible spectra evolution of membrane Mb1, plasticized with oNPOE and doped with 1 wt.% CoTPP-N (without any ion-exchanger sites’ addition), soaked first in 0.01 M aqueous solution of NaCl (red line) for 48 h and then in 0.01 M NaNO_2_ (green line) for 2 h. For comparison, the UV-visible spectrum of fresh Mb1 soaked in 0.01 M NaNO_2_ (blue line) for 2 h and then conditioned in 0.01 M NaCl for 24 h is also shown.

Kinetic studies were carried out by following the increase of the Soret band intensity at 437 nm with time. UV-visible spectral changes of CoTPP-N (5 × 10^−6^ M) upon aggregation in the presence of 20 times excess of TBNO_2_ (1 × 10^−4^ M) are shown in Figure 2. A typical absorbance vs. time profile is reported in the inset 1 of Figure 3. The experimental decays can be well fitted by a stretched exponential kinetic, which is described by following Equation (1):

*E = E_0_ + (E_inf_ − E_0_)exp[−(kt)^n^]* (1)

where *E, E_0_, and E_inf_* are the values of extinction at time *t*, initially, and at equilibrium, respectively. The kinetic parameters, *k* and *n*, were obtained by nonlinear least-squares regression fit to Equation (1) and are reported in Table 2 and insets 1,2 of Figure 2.

From the data shown in Table 2, and in inset 2 in Figure 2, the kinetic behavior of NO_2_^−^-triggered CoTPP-N aggregation can be interpreted on the basis of an apparent first-order regime, deriving from a complex mechanistic path, in which a fast pre-equilibrium of anion exchange (Cl^−^/NO_2_^−^) was followed by a slow aggregation process, as described by Equation (2):

(2) $$\mathrm{Co}TPP\text{-}N^{+}Cl^{-}+{\mathrm{NO}}_{2}^{-}\begin{array}{c} \overset{K_{ex}}{\to }\\ \underset{fast}{\leftarrow } \end{array}\left[\mathrm{CoT}\mathrm{PP}\text{-}N^{+}{\mathrm{NO}}_{2}^{-}\right]\begin{array}{c} \overset{k_{agg}}{\to }\\ slow \end{array}{\left[CoTPP\text{-}N^{+}/NO_{2}^{-}\right]}_{m}$$

In other cases, characterized by a diffusion limited aggregation (DLA ) process, the values of the parameter *n* are consistently found to be <1, which indicates the formation of large aggregates by interaction between initial smaller clusters and monomers, and was previously established for several porphyrinoids’ complexes [30,31]. For our experiments: (1) the aggregation factor *n,* which is related to the availability of nucleation centers during the aggregate growth, remained virtually unchanged and close to the unitary value, indicating the occurrence of a “reaction-limited” pseudo-first-order kinetic regime and (2) the overall aggregation rate (*k_app)_* was found to be dependent on analyte concentration in solution, spanning over 1.5 orders of magnitude in increasing salt concentration, with a clear saturation effect, corroborating the mechanistic path proposed. 

**Scheme 2:**
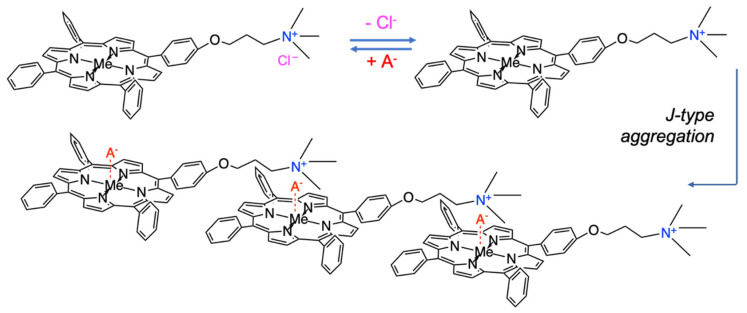
Proposed mechanism of CoTPP-N aggregation in ACN solution in the presence of A^−^ target anions.

An interpretation on the obtained kinetic studies’ results brought us to the conclusion of possible ligand–analyte interaction mechanism, which included at the first step the CoTPP-N ionization and Cl^−^ ions’ release in solution, followed by the axial coordination of one primary anion A^−^ on co-center of CoTPP-N and formation of a kind of ‘zwitterion’ ligand particles, bearing both positive and negative charges on their opposite sides. Finally, the subsequent electrostatic interaction among ligand/NO_2_^−^ particles through the positively charged tris-methylammonium fragment resulted in the formation of head-to-tail, J-type porphyrin aggregates (Scheme 2).

### 3.2. Spectroscopic Tests and Anionic Sensitivity of Co(II)-5-[4-(3-Trimethylammonium)-Propyloxyphenyl]-10,15,20-Triphenyl-Porphyrin Chloride-Based Membranes

The aggregation of CoTPP-N promoted in the presence of an analyte anion gave us an idea of possible application of amphiphilic porphyrinic ligands for anion-sensitive sensors’ development. We, hence, investigated the properties of a PVC-based solvent polymeric membrane doped with CoTPP-N in order to check out the possibility of a ligand-analyte co-aggregation inside a lipophilic polymeric phase. To promote the mobility of active sites inside the membrane, the polar o-nitrophenyl octyl ether (oNPOE, ε_oNPOE_ = 24) was selected as a plasticizer for a membrane Mb1, doped with 1 wt.% of CoTPP-N and prepared without any lipophilic ionic additives. (See Table 1 for membrane compositions). The cocktail of membrane Mb1 in THF was deposited by drop-casting method on the glass transducer surface, and the membrane UV-visible absorbance spectra in aqueous solutions in the absence and in the presence of target nitrite anion were registered (Figure 3). As demonstrated in Figure 3, a formation of a small shoulder at 437 nm on the initial Soret band at 413 nm, corresponding to CoTPP-N monomer, indicated some partial ligand association after two days of newly prepared membrane Mb1 soaking in 0.01M NaCl solution. Upon transfer of the same membrane in NaNO_2_ solution of equal concentration, the formation of a single Soret band at 437 nm and complete disappearance of monomer band at 413 nm were registered already after 2 h of membrane soaking. Such a behavior indicated a complete CoTPP-N ligand association inside a polymeric membrane phase promoted by the nitrite ion. Moreover, for the freshly prepared membrane Mb1 (blue line in Figure 3), the CoTPP-N/NO_2_^−^- aggregates formed during initial 2 h soaking in 0.01 M NaNO_2_ remained unchanged even after subsequent membrane conditioning in 0.01 M NaCl for 24 h. Obtained results indicated the higher affinity of CoTPP-N ligand to the nitrite ions (in comparison to Cl^−^- counter ions) and confirmed our hypothesis on membrane selective behavior through ligand-analyte co-aggregation inside polymeric membrane phase.

**Figure 4:**
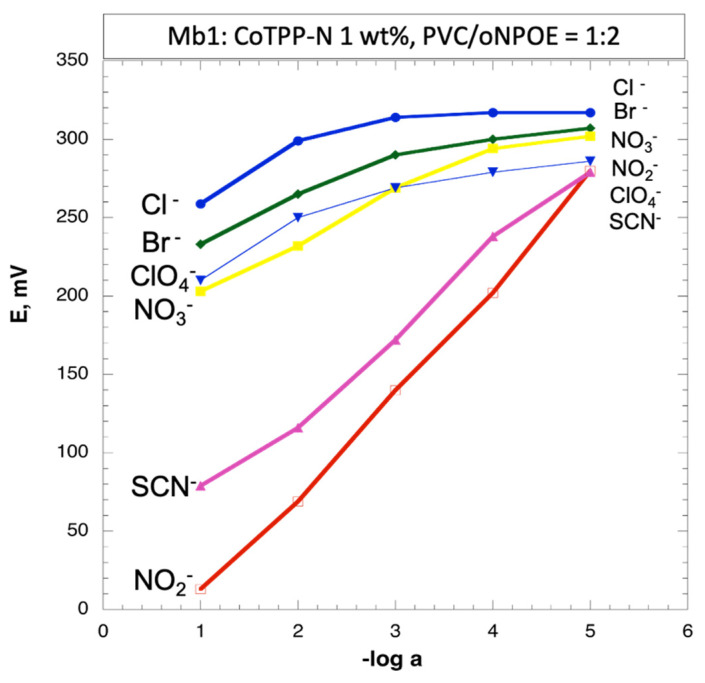
Calibration curves of PVC/o-NPOE membrane Mb1 doped with 1 wt.% of CoTPP-N without any ion exchanger addition. Measurements in 0.01 M MES buffer solution, pH 5.5.

At the next step, potentiometric experiments were performed for CoTPP-N doped membrane Mb1. As can be seen in Figure 4, CoTPP-N membrane Mb1, prepared without any ion-exchanger sites’ addition, showed an enhanced selectivity toward nitrite ion in comparison to all other tested anions. 

The response curve of the membrane Mb1 toward nitrite was linear in all the tested concentration ranges with a slightly super-Nernstian slope of −66.7 ± 2.3 mV/dec. The anti-Hofmeister selectivity pattern of NO_2_^−^ > SCN^−^ > NO_3_^−^ > ClO_4_^−^> Br-> Cl- was observed for membrane Mb1. The obtained selectivity coefficients, log K^pot^_NO_2___/X,_ (Cl^−^, –3.83; NO_3_^−^, –3.27; Br^−^, –3.6; ClO_4_^−^, –3.02; SCN^−^, –0.78) were lower than those of commercially available nitrite ionophore I, based on co-correlate B_12_ derivative, reported in literature [3] (Cl^−^, –3.7; NO_3_^−^, –2.72; Br^−^, –3.3; ClO_4_^−^, –2.4; SCN^−^, 0.2), thus indicating the high potential of CoTPP-N ligand to be used as a selective ionophore for nitrite-selective potentiometric electrodes. 

Unfortunately, membrane Mb1 showed a low reproducibility due to the fast ionophore crystallization: The day after the first measurements, a complete response deterioration was registered. Evidently, an addition of lipophilic ionic sites inside the CoTPP-N-doped membrane Mb1 is required in order to stabilize its response and to prolong a lifetime. Previously, it was shown that the addition of lipophilic ionic additives in porphyrinoids-based polymeric membranes is critical for both the stabilization of membrane properties and the optimization of selectivity [32,33]. Depending on the working mechanism of the anion-selective ionophore, cationic additives are required to neutralize a negatively charged ionophore-anion complex in case of neutral carrier mechanism, while an addition of anionic lipophilic sites will stabilize the charged form of the ionophore (charged carrier mechanism due to ionophore dissociation or protonation). Hence, an additional insight on ionophore functioning mechanism and ionophore-anion complex stoichiometry can be done through addition in the membrane phase of different (cation or anion) ion exchangers in different molar ratios (or different percentages) in respect to the ionophore amount. Such experiments were further performed for CoTPP-N-doped membranes and are described in the sections below. 

### 3.3. The Potentiometric Properties of the CoTPP-N Membranes with Lipophilic Sites and Membranes’ Sensitivity Simulation 

Various amounts of both cationic and anionic lipophilic additives were added inside the CoTPP-N-doped membranes, plasticized with oNPOE in order to investigate ionophore working mechanism and optimize the sensor response characteristics, including the lifetime. Compositions of tested membranes are listed in Table 1. New PVC/oNPOE membranes based on CoTPP-N and/or CoTPP ligands and containing anionic (TpClPBK) or cationic (TDMACl) lipophilic additives, in the amount varying from 20 to 80 mol% relative to the ionophore, were tested and their responses were compared to the response of only anion exchanger-based membrane Mb8. 

**Figure 5:**
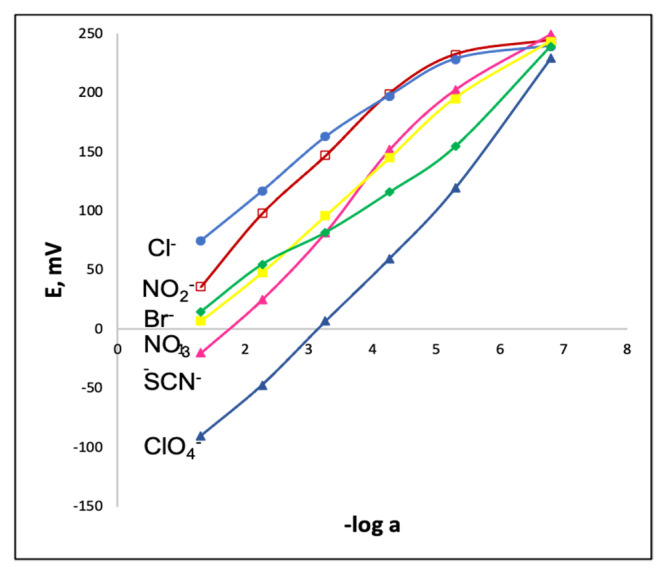
Calibration curves for membrane Mb2 containing CoTPP-N and 20 mol% of TDMACl. Measurements were performed in MES buffer background, pH 5.5.

As can be seen in Figure 5, the membrane Mb2, doped with 20 mol% of TDMACl cationic additive, showed a partial anionic response toward NO_2_^−^ ions with a selectivity closely resembling Hoffmeister pattern. Also, the slope of the membrane Mb2 calibration curve toward the nitrite ion changed significantly over a one-month period of experiments: from –36.5 ± 3.1 mV/dec for freshly prepared membrane. The slight improvement was registered after two weeks of membrane exploitation (−42.5 ± 2.7 mV/dec), followed by significant decrease to –16.8 ± 2.1 mV/dec after four weeks (data are not shown). Such a behavior indicated the prevalent functioning of membrane through anion-exchange mechanism on TDMA^+^ sites, while the CoTPP-N ionophore was present mainly in associated form. The processes of ion pairs’ association/dissociation, accomplished by the ionophore-analyte co-aggregation, may be a result of some improvement in nitrite sensitivity after two weeks of exploitation and of a longer sensor lifetime, in comparison to the membrane Mb1 (prepared without lipophilic sites’ addition). 

**Figure 6:**
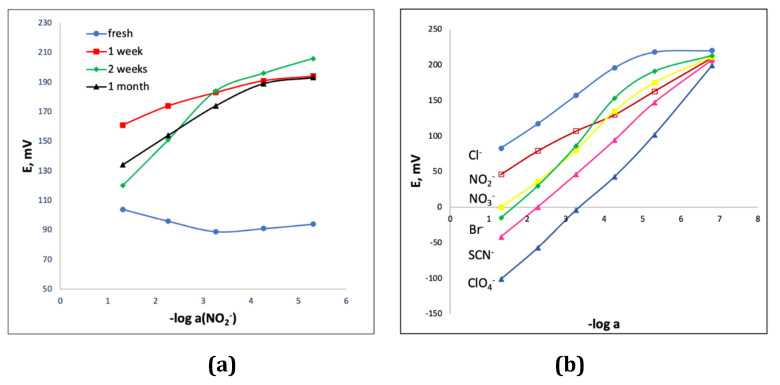
Calibration curves of CoTPP-N-based (**a**) membrane Mb4 doped with 80 mol% of TpClPBK in NaNO_2_ solutions over one-month period of continuous tests; (**b**) membrane Mb3 containing CoTPP-N and 20 mol% of TpClPBK in sodium salts of several anions. Measurements were performed in MES buffer background, pH 5.5.

The addition in membrane Mb4 of anionic TpClPB^−^ sites in the amount close to the ionophore content (1:0.8 ratio) resulted in the membranes’ cationic response, determined by the initial excess of cation exchanger. Interestingly, over the one-month period of membrane Mb4 testing, its response inside NaNO_2_ solution deteriorated, passing from the partial cationic response with a slope of 10.5 ± 5.2 mV/dec to the partial anionic sensitivity after two weeks (−22.8 ± 2.2 mV/dec), with a further slope decrease to the value of −11.3 ± 3.1 mV/dec after one month from the tests’ start (Figure 6a). 

The partial cationic response may have been caused by cation exchanger (TpClPB^−^) and dissociated ionophore (CoTPP-N^+^) association, thus resulting in excess of overall anionic sites inside a membrane and Na^+^ co-ion interference. Over time, the CoTPP-N^+^/TpClPB^−^ ionic pairs inside a membrane are going to dissociate due to the higher CoTPP-N affinity to nitrite ions, thus also resulting in increase of a partial anionic response. Finally, after one month from the tests’ start, the process of partial aggregates’ crystallization may have been a reason for anionic slope decrease. 

For membrane Mb3 containing 20 mol% of TpClPBK, the stable partial anionic response toward NO_2_^—^ ions with a mean slope of −40.3 ± 1.7 mV/dec was observed over a one-month period, except the first calibration of the fresh membrane, for which the lower slope of −25.7 ± 3.2 mV/dec was registered. This behavior was in accordance with our previous assumption on initial partial cation exchanger/ionophore association in a fresh membrane: Due to the low TpClPB- sites’ amount (20 mol% in respect to ionophore), the dynamic equilibrium among ionized species inside membrane was further reestablished in the presence on primary nitrite ions; however, due to the excess of CoTPP-N+ cationic anion-exchanging sites, a selectivity close to classical Hofmeister selectivity series was observed (Figure 6b). 

In order to explain an obtained Hoffmeister selectivity pattern for CoTPP-N membranes Mb2–Mb4 doped with anionic and cationic lipophilic sites, the membrane sensitivity was simulated numerically with no assumptions on the association degree of any type of the species present in the membrane phase. Two limiting cases were considered: case 1, the classical ISE membrane doped with MeTPP ionophore and 20 mol% of anion exchanger in respect to ionophore; case 2, the above-discussed membrane Mb1, containing only MeTPP-N ligand. Moreover, a third case, considering a membrane based on metalloporphyrin ionophore MeTPP and doped with about 20 mol% of MeTPPN, which in this case played mainly an anion-exchanger role, was investigated. 

From kinetics studies it was confirmed that the co-aggregation between (except for case 2, considering the full CoTPP-N aggregation) ligand and the nitrite anion was a process, dependent on analyte concentration in solution, while the stoichiometry of initially formed ionophore-anion ion pairs was 1:1. The further formation of larger aggregates could be interpreted through interaction between initial smaller [CoTPP-N/NO_2_^−^]*_m_* clusters and monomers. Also, to remain in solution without crystallization, the aggregates’ size should not exceed the *m* ~ 20−25 aggregated ionophore molecules, while for lipophilic membrane phase this number should be even smaller (*m* ~ 2−5) to avoid ionophore precipitation. For simplicity, it was assumed that in the membrane phase all the single ionic species (such as primary ion I^−^, interfering ion J^−^, and anion exchanger R^+^) were monovalent. Also, the CoTPP-N^+^ was initially present as monovalent positively charged species formed through ionophore dissociation, accomplished by Cl^−^ ions leaching outside the membrane. No numerical values were assigned to the association constants (K^ass^_X_) between CoTPP-N^+^ and anions, but it was silently assumed that due to the high affinity of Co-porphyrins to NO_2_^−^ ions, the ratio K^ass^_I_/K^ass^_J_ was a high and a constant value. Moreover, in case 3, when CoTPP macrocycle was incorporated inside the composition of polymeric solvent membrane, it was considered to function as a neutral carrier. The simplificated estimation of sensor sensitivity was performed as a direct ratio of selective interactions of primary ion I^−^ and non-selective interactions of interfering ion J^−^ with membrane-active sites. Table 3 summarizes the results of sensitivity estimations. As it may be observed from the Table 3, the best sensitivity toward primary NO_2_^−^ ion (except the case 2, considering the full CoTPP-N aggregation) was found in case 3 for a membrane doped with neutral CoTPP ionophore and 20 mol% of CoTPP-N that played a role of anion exchanger and considering a partial CoTPP-N self-aggregation. 

### 3.4. Incorporation of Co(II)- 5-[4-(3-Trimethylammonium)Propyloxyphenyl]-10,15,20-Triphenyl-Porphyrin Chloride as a Cation Exchanger in Membranes Based on CoTPP

**Figure 7:**
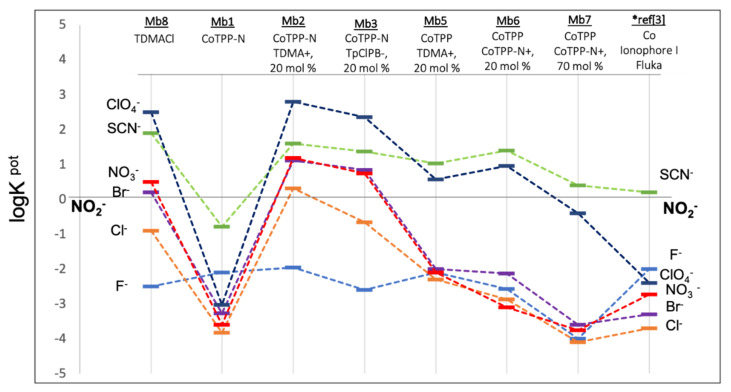
Selectivity coefficients values, logK_NO__2/J_^pot^, of tested membranes containing 1 wt.% of ionophore (CoTPP-N or CoTPP) and various amount of TDMACl and TpClPBK (indicated as mol% relative to ionophore content). All measurements were carried out in MES buffer, pH 5.5. The slope of –57.8 mV/dec was used for calculus. For comparison, the selectivity of membrane Mb8 formulated only with 10 wt.% TDMACl is also shown.

The sensitivity numerical simulations were in a good agreement with experimentally determined potentiometric selectivity coefficients (shown in Figure 7). As expected, among all tested membranes containing CoTPP-N ligand, the best selectivity, significantly different from Hoffmeister pattern (demonstrated by TDMACl-based membrane Mb8), was obtained for membranes Mb6 and Mb7, containing CoTPP with 20 mol% and 70 mol% of CoTPP-N, respectively. The only influence of the highly lipophilic SCN^−^ and ClO_4_^−^ ions on nitrite response was registered, with the response toward SCN^−^ ion stronger, that is, accordance to the higher affinity of co-porphyrins to the species bearing nitrogen atoms.

**Figure 8:**
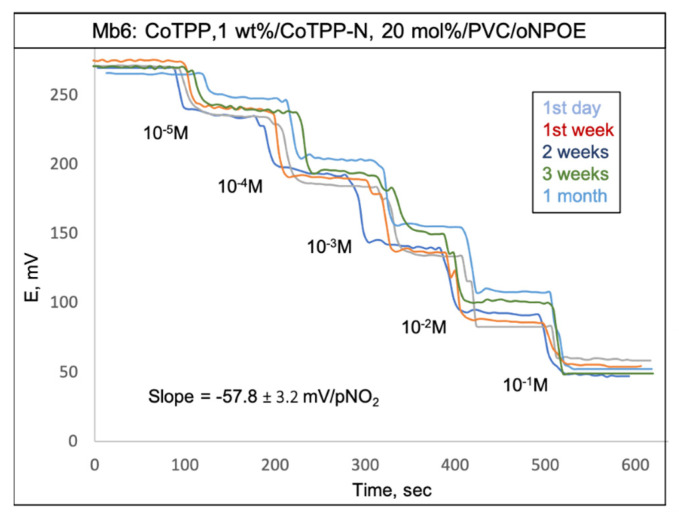
Dynamic response curves of electrodes with membrane Mb6 containing 1 wt.% of CoTPP and 20 mol% of CoTPP-N in NaNO_2_ solutions through one month of exploitation. Measurements were carried out in MES buffer, pH 5.5.

The membrane Mb1 doped with only CoTPP-N showed the similar selectivity toward nitrite ions, but a very short lifetime due to the [CoTPP-N^+^/NO_2_^−^]*_m_* ligand-analyte co-aggregates’ crystallization from the membrane phase. The similar behavior, due to the predominating role of co-aggregation, was registered for membrane Mb7, based on CoTPP ionophore and containing 70 mol% of CoTPP-N. On the contrary, the stability and lifetime of CoTPP/CoTPP-N-based membrane Mb6 were improved significantly. Thus, after one day and one, two, and four weeks of exploitation, the dynamic response curve toward nitrite ions for the membrane Mb6 remained apparently univariate (Figure 8). The mean value of potentiometric response slope calculated over a one-month period was −57.8 ± 2.4 mV/dec, close to theoretical Nernstian slope with a standard deviation of 3.2 mv and LDL estimated as 37 mM. 

## 4. Conclusions

In this work, an application of self-aggregating macrocycle bearing incorporated anion-exchanger fragment, 5-[4-(3-trimethylammonium)propyloxy-phenyl]-10,15,20-triphenylporphyrinate of Co(II) chloride, CoTPP-N, as a sensitive ligand in solvent polymeric membrane electrodes was investigated for a first time. Kinetic and spectroscopic studies permitted us to establish a plausible ligand–analyte interaction mechanism, involving anion-triggered CoTPP-N aggregation, which can be interpreted on the basis of an apparent first-order regime, deriving from a complex mechanistic path, in which a fast pre-equilibrium of anion exchange was followed by a slow aggregation process. Polymeric membranes prepared with CoTPP-N showed enhanced sensitivity toward nitrite ion, and their response characteristics were dependent on the presence of additional membrane-active components (lipophilic ionic sites and ionophore) in the membrane phase. In particular, the possibility of selectivity tailoring and membrane lifetime prolongation was shown through an addition of Co(II)-5,10,15,20-tetraphenylporphyrin, CoTPP, neutral ionophore, while the CoTPP-N was present in an amount of 20 mol% (in respect to the CoTPP) and mainly acted as an anion exchanger. Numerical simulations of CoTPP-N-containing membranes’ sensitivity were in a good agreement with experimental results. Finally, the optimum composition of PVC-based, solvent polymeric membrane nitrite-selective electrode, with a slope of −57.8 ± 3.2 mV/dec, in the concentration range from 10^−6^ to 5*10^−2^ M, LDL of 37 µM, and reproducible performance for more than a one-month period, was developed. The promising results on electrochemical response of obtained sensing membranes are certainly worthy of further studies on MeTPP-N-based sensors’ development, just in progress in our laboratories.

## Figures and Tables

**Table 1:** The compositions of tested CoTPP-N membranes.

Membrane N	Ionophore, 1 wt.%	Additive, mol %	
		TDMA+	TpClPB-
1	CoTPP-N	-	-
2	CoTPP-N	20%	-
3	CoTPP-N	-	20%
4	CoTPP-N	-	80%
5	CoTPP	20%	
6	CoTPP	CoTPPN, 20%	-
7	CoTPP	CoTPPN, 70%	-
8	-	TDMACl, 10 wt.%	-

**Table 2:** Kinetic parameters for the aggregation of CoTPP-N (5.0 × 10^−6^ M) in the presence of NO_2_^−^ ions.

Entry	[TBANO_2_], M	*k_app_*, min^−1^	*n*
1	1.0 × 10^−5^	2.6 × 10^−4^	0.83
2	5.0 × 10^−5^	2.2 × 10^−3^	0.99
3	1.0 × 10^−4^	3.4 × 10^−3^	0.91
4	5.0 × 10^−4^	3.9 × 10^−3^	0.96

**Table 3:** Sensitivity parameters estimated for CoTPP and CoTPP-N-based membranes, considering single-molecule interactions and/or ligand aggregation involved in the membrane response mechanism.

Membrance Composition	Schematic Presentation	Possible Interactions	$\mathrm{Sensitivity}=\frac{I\;interactions}{J\;interactions}$
Case 1: MeTTP ionophore+ 20 mol% ion exchanger	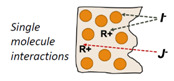	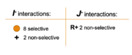	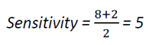
Case 2: MeTTP-N ionophore	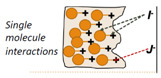	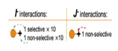	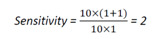
	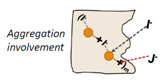	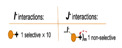	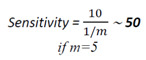
Case 3: MeTTP ionophore + 20 mol% MeTTP-N ion exchanger	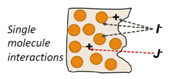	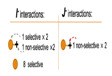	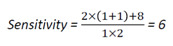
	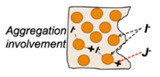	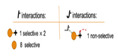	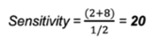

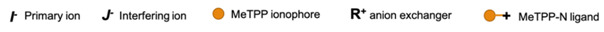

## Data Availability

Not applicable.
